# Comprehensive biomarker analysis from phase II study of nivolumab in patients with thymic carcinoma

**DOI:** 10.3389/fonc.2022.966527

**Published:** 2023-01-09

**Authors:** Yuki Katsuya, Shigehisa Kitano, Makiko Yamashita, Mayu Ouchi, Shigehiro Yagishita, Akinobu Hamada, Hiromi Nakamura, Fumie Hosoda, Tatsuhiro Shibata, Noriko Motoi, Takayuki Nakayama, Takashi Seto, Shigeki Umemura, Yukio Hosomi, Miyako Satouchi, Makoto Nishio, Toshiyuki Kozuki, Toyoaki Hida, Yuichiro Ohe, Hidehito Horinouchi

**Affiliations:** ^1^ Department of Thoracic Oncology, National Cancer Center Hospital, Tokyo, Japan; ^2^ Department of Experimental Therapeutics, National Cancer Center Hospital, Tokyo, Japan; ^3^ Advanced Medical Development Center, The Cancer Institute Hospital of JFCR, Tokyo, Japan; ^4^ Department of Pharmacology and Therapeutics, Fundamental Innovative Oncology Core, National Cancer Center Research Institute, Tokyo, Japan; ^5^ Division of Molecular Pharmacology, National Cancer Center Research Institute, Tokyo, Japan; ^6^ Division of Cancer Genomics, National Cancer Center Research Institute, Tokyo, Japan; ^7^ Division of Genome Biology, National Cancer Center Research Institute, Tokyo, Japan; ^8^ Department of Pathology, Saitama Cancer Center, Saitama, Japan; ^9^ Department of Urology, Saitama Medical Center, Saitama, Japan; ^10^ Department of Thoracic Oncology, National Hospital Organization Kyushu Cancer Center, Fukuoka, Japan; ^11^ Department of Thoracic Oncology, National Cancer Center Hospital East, Chiba, Japan; ^12^ Department of Thoracic Oncology and Respiratory Medicine, Tokyo Metropolitan Cancer and Infectious Diseases Center Komagome Hospital, Honkomagome, Tokyo, Japan; ^13^ Department of Thoracic Oncology, Hyogo Cancer Center, Hyogo, Japan; ^14^ Department of Thoracic Medical Oncology, The Cancer Institute Hospital of Japanese Foundation for Cancer Research, Tokyo, Japan; ^15^ Department of Thoracic Oncology and Medicine, National Hospital Organization Shikoku Cancer Center, Ehime, Japan; ^16^ Department of Thoracic Oncology, Central Japan International Medical Center, Gifu, Japan; ^17^ Department of Thoracic Oncology, Aichi Cancer Center, Aichi, Japan

**Keywords:** thymic carcinoma, nivolumab, immunogenicity, biomarker, peripheral blood mononuclear cell (PBMCs)

## Abstract

In a phase II trial of nivolumab in advanced thymic carcinoma (UMIN000022007), long SD (SD for more than 24 weeks) was seen in three patients and irAE (Gr2 or higher) was seen in four patients among 15 patients. Here, we report preplanned comprehensive biomarker analyses. We obtained tumor samples for immunohistochemistry, peripheral blood mononuclear cells (PBMCs), plasma and serum for pharmacokinetic analysis of nivolumab and cytokine evaluations, and whole blood for immuno pharmacogenomic (PGx) analysis. PD-L1 expression on tumor cells were not associated with therapeutic efficacy, but FOXP3 expression in tumor area and stroma, CD204 expression in stroma, and MHC class I in tumor area were all low among long SD patients. PBMC of long SD patients presented with larger number of naïve/memory cells prior to treatment, suggesting priming after nivolumab administration. Immuno-PGx analysis showed non-synonymous SNVs in *ITGAX* and *PDCD1* had some correlation with PFS. Concentration of nivolumab in blood during the treatment was not related to PFS, with their overall trend towards decreased nivolumab concentration in patients with irAEs. Low immunogenicity of thymic carcinoma demonstrated in our study may require the activation of immune systems *via* a combination of immune checkpoint blockades.

## 1 Introduction

Thymic carcinoma is a rare type of cancer with limited treatment options. However, given that some thymic carcinoma cells express PD-L1, which is used as a treatment biomarker of anti-PD-1 antibody in certain cancer types, we designed and conducted a single-arm, multicenter, phase II trial of nivolumab for the treatment of unresectable or recurrent thymic carcinoma: the PRIMER study (UMIN 000022007) (1).

Biomarkers such as PD-L1 expression, high TMB, and increased tumor infiltration of CD8+ T cells all correlate with the therapeutic efficacy of PD-1 antibodies, but none of these are sufficient to precisely select patient populations. TMB, in particular, varies widely in its degree of association with ICI efficacy and is largely dependent on cancer type (2).

The PRIMER study revealed a durable clinical benefit, defined as stable disease (SD) for over 24 weeks (long SD +), in 3 out of 15 patients, despite the presence of an early termination criterion (less than one responder) in the first stage of the study. Immune-related adverse events (grade 2 or higher, related with nivolumab) was seen in four patients. Patients were required to provide tumor tissue and blood samples at several points, pre-, during, and post-treatment, to facilitate a later biomarker study.

To date, there has been little comprehensive immunological analysis of thymic carcinoma. In a recent phase 2 trial of pembrolizumab for patients with thymic carcinoma, immunohistochemical (IHC) positivity for PD-L1 demonstrated a significant correlation with treatment response. In another study, a genetic panel test of these samples revealed that increased expression of T cell-flamed IFNγ also correlated with treatment response (3).

Here, we report the results of a comprehensive immunological evaluation of both the tissue and blood samples from the PRIMER study and identify several potential biomarkers for future evaluation.

## 2 Materials and methods

### 2.1 Patients

All of the patient eligibility criteria for the PRIMER study are as previously described (1). Briefly, enrolled patients had to meet the following criteria: (1) unresectable or recurrent thymic carcinoma (regardless of PD-L1 expression), (2) Eastern Cooperative Oncology Group-performance status of 0 or 1, (3) progression after at least one round of chemo(radio)therapy, and (4) no history of autoimmune disease. All enrolled patients provided written informed consent for their participation.

This study was conducted as an open-label, two-stage, single-arm, multicenter, phase II trial assessing the efficacy and safety of nivolumab (3 mg/kg intravenously every 2 weeks until disease progression) (UMIN000022007), and was approved by the National Cancer Center Hospital (NCCH 2016-145), in accordance with the ethical guidelines set out in the Declaration of Helsinki.

### 2.2 Immunohistochemistry (IHC) of single staining on tissue samples

Single target IHC was performed using tissue samples. This staining was completed using 5 μm-thick sections of the formalin-fixed paraffin-embedded (FFPE) tissues. All specimens were obtained through biopsy, except for one (#13) from surgical resection. We then evaluated the antigenicity of the tumor and surrounding environment using antibodies against PD-L1 (expression on cytoplasm + membrane of tumor cells, clone 28-8), CD8 (membrane of T cells, 4B11), FOXP3 (cytoplasm of Tregs, 236A/E7), CD204 (membrane of M2 macrophages, SRA-06), PD-1 (cytoplasm of TILs, NAT105), VEGR2 (membrane of tumor cells, FLK-1), MHC class1 (cytoplasm + membrane of tumor cells, EMR8-5), MHC class2 (cytoplasm + membrane of tumor cells, CR3/43), CD20 (cytoplasm + membrane of B cells, L26). The number of positive immune cells at the hot area were visually quantified and calculated the number per 1 mm squares from both the tumor and stromal regions, respectively. The percentage of positive tumor cells was scored independently by two observers, including a well-experienced pathologist (YK, NM).

### 2.3 Multiplex fluorescence immunohistochemistry (mFIHC) on tissue samples

These evaluations used 5 μm sections of the FFPE tissues described above and mFIHC staining *via* the Opal Kit (AKOYA Biosciences, California, USA) to determine the cross reactivity of these tumors and their adjacent tissues. The antibodies, dilutions, and activation conditions are listed in **Table S1**. Slides were scanned using a Vectra slide scanner (AKOYA Biosciences) and the mean fluorescence intensity for each biomarker in each case was then used as the base from which the software could identify positive calls. In the case of the multispectral analysis, each individually stained section was used to establish a spectral library for each of the fluorophores and between 5 and 20 random areas from each sample were blindly reviewed and analyzed by two pathologists at 20× magnification.

This analysis was developed using previously reported methods^19,20^. An image analysis program (Inform; AKOYA Biosciences) was used to segment tumor tissues into carcinoma and stromal areas, and then individual immune cells, with defined phenotypes, where identified and their distribution evaluated. Training sessions for tissue segmentation and phenotype recognition were also conducted (**Figure S1**). We then used the phenotypes of typical CD4^+^ and CD8^+^ cells as defined by the Inform software to identify these subpopulations within the larger CD3^+^ cell population. A similar gating strategy was also used to identify Foxp3, Ki-67, PD-1, and Tim-3 populations in both the CD4^+^ and CD8^+^ T cell cohorts using Spotfire version 7.8 TIBCO Software, CA, USA). We then calculated the area of each tissue category within the intratumoral and peritumoral areas and used this to determine the density of each immune cell type, as described below:


$$Density\;of\;immune\;cells\;=\;immune\;cell\;count/intratumoral\;or\;peritumoral\;area\;(mm^{2})$$

T cells within the intratumoral and peritumoral areas were defined as intratumoral or peritumoral T cells, respectively.

### 2.4 Isolation of peripheral blood mononuclear cells (PBMCs)

Peripheral blood was collected at baseline, Cycle 3 Day 1 (C3D1), C5D1, C9D1, and on progression disease (PD) (when available), and then centrifuged using Histopaque-1077 (Sigma Aldrich, St Louis, MO, USA) as previously described. Briefly, the samples were diluted with PBS and layered on Histoaque-1077 before being centrifuged at 800 × g for 15 min at room temperature. Mononuclear cell components were collected, washed twice with PBS, and stored at -80°C in CELLBANKER I (Takara Bio Inc. Shiga, Japan). These PBMCs were then frozen in liquid nitrogen until use.

### 2.5 Definition and analysis of immune cell subsets

We then thawed our cryopreserved PBMCs and washed them twice in PBS supplemented with 10% FBS. Single-cell suspensions were then processed for surface staining with an antibody cocktail (panel described in **Table S2**) for 20 min at 4°C and then washed with PBS containing 2% FBS, fixed, and permeabilized using the Foxp3 Staining Buffer Set (BD Biosciences, Franklin Lakes, NJ, USA) according to the manufacturer’s instructions. Cells were then stained with an intracellular antibody cocktail (panel described in **Table S2**) for 30 min at 4°C before being washed in Foxp3 permeabilization buffer and resuspended in CellFix (BD Biosciences). The stained cells were then evaluated using an LSR II Fortessa with FACS Diva software (BD Biosciences) and all analyses were completed using FlowJo software (BD Biosciences, Franklin Lakes, NJ, USA).

### 2.6 Pharmacokinetic analysis of nivolumab

We determined the serum nivolumab concentration (trough values) of 15 samples using high-performance liquid chromatography/tandem mass spectrometry (LC-MS/MS) and serum samples from Pre, C3D1, C5D1, C9D1, and PD (when available) as previously described (4).

### 2.7 Serum cytokine evaluations

We evaluated the cytokine expression in these samples using sera from Pre, C3D1, C5D1, C9D1, and PD (when available) and the Multiplex Human Cytokine/Chemokine Panel evaluating IFNγ, IL-1, IL-7, TNFα, MIP-1a, MIP-1b, IL-6, IL-17A, IL-5, IL-10, IL-12p40, IL-1b, IL-8, IP-10, and MCP-1 (Millipore Corporation, MA, USA). Each cytokine was evaluated in duplicate.

### 2.8 Immuno Pharmacogenomic (PGx) analysis from blood samples

High-molecular-weight DNA was extracted from whole blood samples using the QIAamp DNA Blood Mini Kit (Qiagen, Venlo, Netherlands) and then subject to evaluation of the 117 genes associated with therapeutic response to immunotherapy strategies (NCC_Immuno_PGx Panel, **Table S3**). The capture probes were custom-designed using SureDesign software (Agilent Technologies, Santa Clara, CA, USA) and the target-capture library preparation and sequencing were conducted as previously described (5). Briefly, calls for germline single-nucleotide variants (SNVs)/indel/copy number variation (CNV) were annotated.

### 2.9 Statistical analysis

We used the Mann-Whitney test to compare factors in the long SD (+) and (-) groups, and irAE (+) and (-) groups (GraphPad Prism 9). Fisher’s exact test was used to analyze the correlation between long SD, irAE, SNV, and CNV candidates from the ImmumoPGx analysis and log-rank tests using Kaplan-Meier methods were completed to compare PFS between the two or three groups, respectively.

## 3 Results

### 3.1 Patient characteristics and specimen accumulation

Fifteen patients from five institutions were enrolled in PRIMER study. All were Japanese, 12 were male, the median age was 55 (range 34 to 70), 11 had an ECOG-PS of 1, and 13 had a squamous histology. Among 15 patients, long SD was seen in three patients (#11, #13, and #14) and irAE was seen in four patients (27%, #4, #10, #13, and #14) in PRIMER study. These irAEs included Gr3 AST elevation on C1D8 (#4), Gr2 hypothyroidism on C3D6 (#4), Gr2 diarrhea on C3D10 (#10), Gr2 maculopapular rash on C17D1 (#13), Gr2 oral leukoplakia on C19D15 (#13), Gr2 adrenal insufficiency on C10D8 (#14), and grade 2 lichen planus on C33D8 (#14). We obtained archival tissue specimens from 14 patients (except #8). Case #13 was a surgical specimen, while the other 14 samples were all biopsy specimens. We also obtained whole blood (pre), plasma (pre, C3D1, C5D1, C9D1, PD), and serum (pre, C3D1, C5D1, C9D1, PD) from 15 patients, but PBMCs (pre, C3D1, C5D1, C9D1, PD) were collected from only nine patients (#1,2,3,4,5,6,10,14,15) due to limitations in the PBMC isolation process (**Table 1**). All specimens were then successfully evaluated in our downstream assays.

**Table 1:** Patient characteristics and treatment results from the PRIMER study cohort.

Pt #	Age	F/M	Histology	Stage	ECOG‐PS	Smoking status	Best response	PFS (months)	Long SD	irAE (Gr2 or more, related to nivolumab)	Archival tissue	Whole blood,Serum,plasma	PBMC
1	68	M	Sq	IVb	1	current	SD	3.8			yes	yes	yes
2	66	M	Sq		0	ex	SD	3.7			yes	yes	yes
3	55	F	Sq	IVb	1	never	SD	3.7+			yes	yes	yes
4	51	M	Sq	IVb	1	ex	PD	2.1		Gr2 Hypothyroidis m on C3D6	yes	yes	yes
5	47	M	Sq	IVb	1	never	SD	5.6			yes	yes	yes
6	70	M	Sq	IVb	1	ex	PD	1.9			yes	yes	yes
7	37	M	Sq	IVb	0	ex	PD	1.4			yes	yes	
8	55	M	Ad	IIIa	1	ex	SD	2.8				yes	
9	40	F	Sq	IVa	1	never	SD	5.6			yes	yes	
10	40	M	Sq	IVa	1	never	PD	1.9		Gr2 diarrhea on C3D10	yes	yes	yes
11	48	M	Thymic carcinoma, NOS	IVb	1	ex	SD	7	yes		yes	yes	
12	60	F	Sq	IVb	0	ex	SD	3.8			yes	yes	
13	34	M	Sq	IVb	1	never	SD	11.3	yes	Gr2 Rash maculopapula r on C17D1, Gr2 leukoplakia on C19D15	yes	yes	
14	67	M	Sq	IVb	1	ex	SD	16.8+	yes	Gr2 renal insufficiency on C10D8, Gr2 lichen planus on C33D8	yes	yes	yes
15	63	M	Sq	IVb	0	ex	SD	3.9+			yes	yes	yes

+, censored; Ad, adenocarcinoma; Sq, squamous cell carcinoma; NOS, not otherwise specified.

### 3.2 Pharmacokinetic analysis of nivolumab

**Figure 1:**
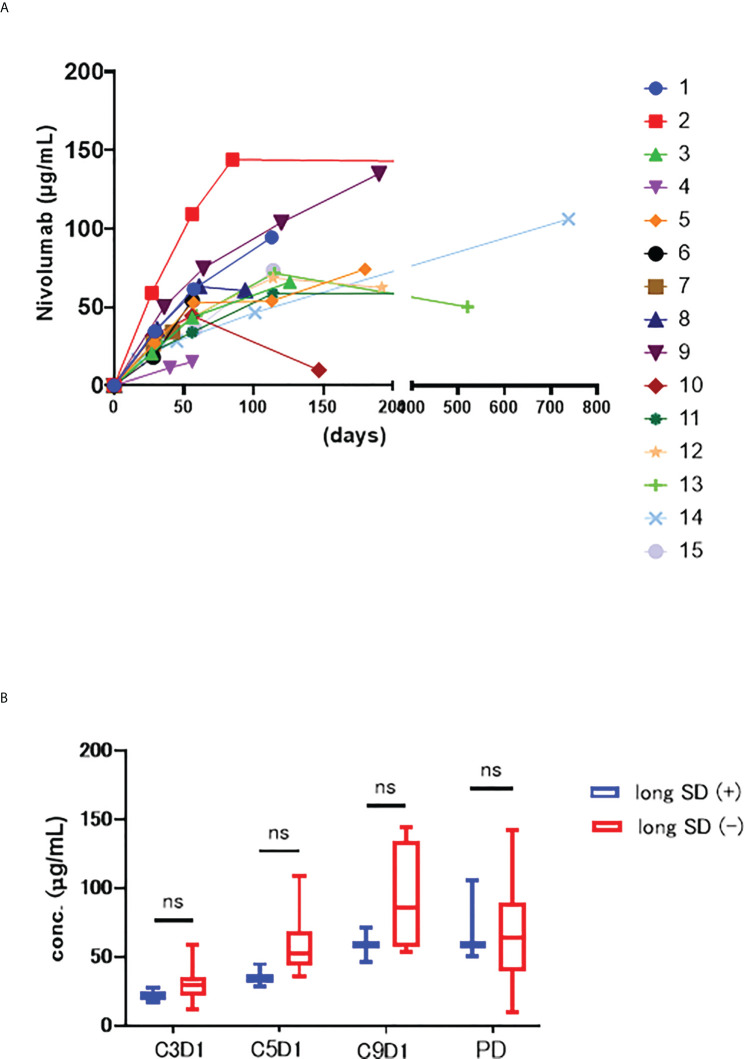
**(A)** Serum concentrations trend of nivolumab in each patient. The horizontal axis shows the number of days since the start of nivolumab administration, and the vertical axis shows the blood concentration of nivolumab. **(B)** The serum concentration of nivolumab at each time point of long SD (+) and long SD(-) groups. ns stands for not significant.

The serum nivolumab concentrations of each sample are shown in **Figure 1A**. The median serum concentration was 68.6 µg/mL (range 15.1–144.0). Despite individual differences, there were no major outliers in these data (6, 7). The large drop in serum concentration in sample #10 was due to a delay in sample collection at PD.

Next, we compared the long SD (+) and (−) groups at each timepoint (C3D1, C5D1, C9D1, and PD) (**Figure 1B**) but noted no significant changes in at C3D1 (p=0.294), C5D1 (p=0.063), C9D1 (p=0.400), or PD (p=0.945), although these concentrations were all relatively low in the long SD (+) group.

### 3.3 Factors associated with PFS

#### 3.3.1 IHC and fMIHC

All 14 specimens contained viable tumor cells, but in two cases (#12, 15) the entire specimen was made up of tumor cells making it impossible to evaluate the stroma in these samples.

Of the three long SD (+) patients two presented with no PD-L1 expression and one with 40% positivity. Of the 11 long SD (-) patients, 5 presented with 0% staining, 1 with 3%, 2 with 10%, 2 with 20%, and 1 with 50% PD-L1 expression.

**Figure 2:**
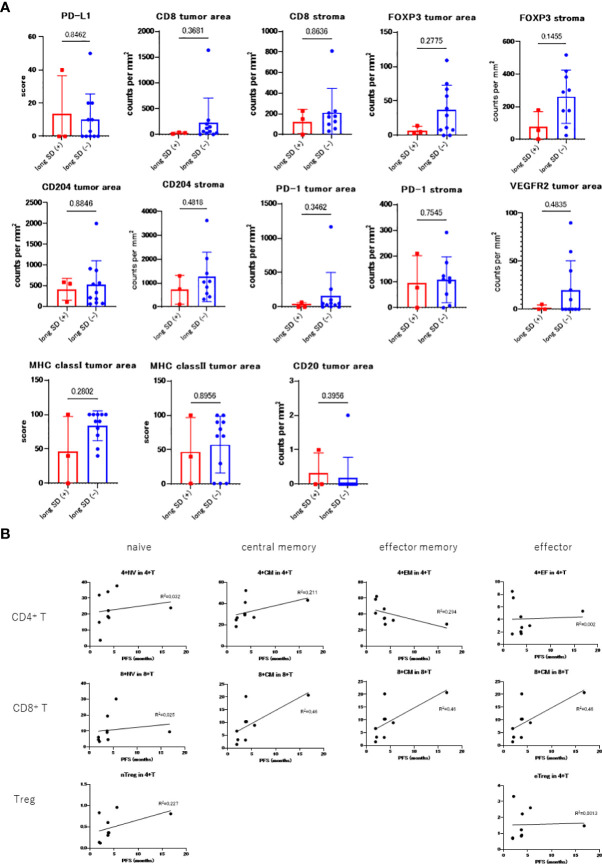
**(A)** Comparing the immunostaining scores between long SD (+) and long SD (-) groups. **(B)** PBMC count in each patient prior to treatment and correlation with PFS.

**Figure 2A** reveals the results of the immunostaining experiments in each of these two groups: long SD (+) and long SD (-) and reveals that the number of tumor-infiltrating CD8^+^ T cells was lower in the long SD (+) group (median 34 vs. 61, p = 0.368). There was only a minor difference in the number of stromal CD8^+^ T cells in these groups (median, 150 vs. 172; p = 0.863). MHC class I expression in the tumor cells (median 40 vs. 90, p = 0.280), FOXP3+ tumor area (median 5 vs. 20, p = 0.277), FOXP3+ stroma (median 56 vs. 290, p=0.145), and CD204+ stroma (median 748 vs. 1049, p=0.481) were all lower in the long SD (+) group.

Multiple staining for CD4, CD8, CD204, and FoxP3 was also performed and the long SD (+) samples were all shown to exhibit significantly lower numbers of CD4+ (1.03E-08 vs. 9.51E-07, p = 0.0092) and CD8+ (1.69E-06 vs. 1.12E-05, p = 0.030) T cell infiltration, as well as a reduced number of CD204+ (5.91E-06 vs. 2.98E-05, p = 0.015) and FOXP3+ (1.04E-08 vs. 4.31E-07, p = 0.0095) cells in the tumor area. However, this pattern was not observed in the stromal areas; CD4+ (6.87E-07 vs 4.2E-06, p = 0.013), CD8+ (8.4E-06 vs 9.58E-06, p = 0.45), CD204+ (3.95E-05 vs 4.81E-05, p = 0.39), and FoxP3+ (6.24E-08 vs 7.41E-07, p = 0.054).

#### 3.3.2 PBMC

The relationship between the PBMC count obtained before treatment and PFS is shown in **Figure 2B**. Samples before treatment with higher numbers of CD4^+^ and CD8^+^ memory cells tended to have higher PFS values. The ratio of the C3D1 and the pre- values for CD80^+^, Ki67^+^ naïve/memory T-cells, CD80^+^, PD-L1^+^ monocytes, and myeloid-derived suppressor cells (MDSCs) positively correlated with PFS (**Figure S1**).

#### 3.3.3 Immuno-PGx analysis

Non-synonymous SNVs in *ITGAX* (rs2230429, C>G) and *PDCD1* (rs2227982, G>A) where shown to have some correlation with PFS (**Figure S3**). In addition, none of these samples presented with *wild-type ITGAX* (rs2230429), six patients had heterozygous mutations, and nine patients had homozygous mutations. The heterozygous group presented with a prolonged PFS (HR 0.27, 95% CI, 0.069 – 1.12, p = 0.067). Five patients also presented with *wild-type PDCD1* (rs2227982), seven had heterozygous mutations, and three had homozygous mutations. The homozygous mutation group showed a longer PFS than the other groups (wild + heterozygous vs. homozygous; HR 0.20, 95% CI 0.039 – 1.02, p = 0.053).

### 3.4 Factors associated with irAEs

#### 3.4.1 PFS

**Figure 3:**
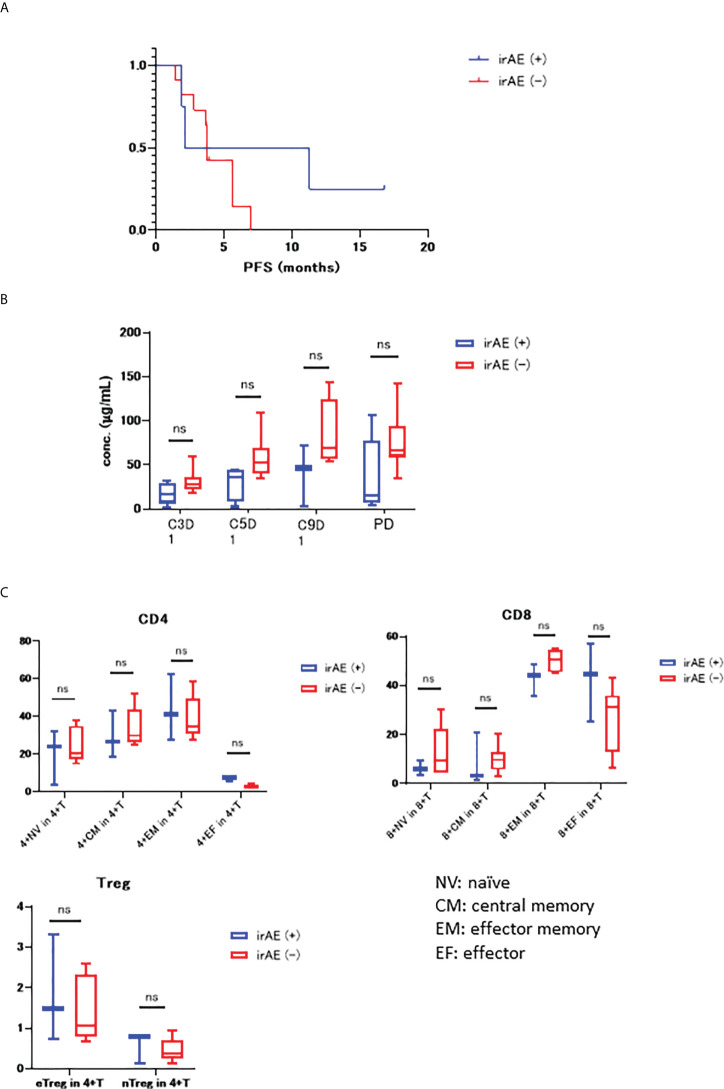
**(A)** KM curve for PFS in both the irAE (+) and irAE (-) groups (mPFS 6.7 months vs 3.8 months, HR 0.56, 95% CI 0.17–1.83, P=0.306); **(B)** Differences in the serum concentration of nivolumab in irAE (+) and irAE (-) groups. **(C)** PBMC count for both irAE (+) and irAE (-) prior to treatment. NV, naïve; CM, central memory; EM, effector memory; EF, effector. “ns” stands for not significant.

We examined the differences in PFS between patients two groups of irAE (+) and irAE (-), which revealed that the mPFS value was 6.7 months in the irAE (+) group and 3.8 months in the irAE (-) group, but this difference was not significant (HR 0.56, 95% CI 0.17 – 1.83, p = 0.306) (**Figure 3A**).

#### 3.4.2 Nivolumab pharmacokinetics

**Figure 3B** shows the differences in the concentrations of nivolumab in the serum samples from irAE (+) and irAE (−) samples, with their overall trend towards decreased nivolumab concentration in the irAE (+) group.

#### 3.4.3 PBMC

We then went on to examine the correlation between PBMC numbers before treatment and irAE status, with these evaluations comparing the CD4^+^ T cells, CD8^+^ T cells, Treg naïve, central memory, effector memory, and effector ratios in the irAE (+) and irAE (-) groups (**Figure 3C**). These experiments revealed that both CD4^+^ and CD8^+^ effector cells were higher in the irAE (+) group.

#### 3.4.4 Cytokines

None of the patients presented with consistently high levels of IFNγ, IL-2, IL-7, TNFα, MIP1α, or MIP1β. There was some increase in these inflammatory cytokines in some of the patients with long SD (#13) and irAEs (#4, 10). IL-10 was found to be characteristically elevated in two cases (#4, 10), in which the irAEs occurred relatively early on in progression but overall, while the cytokine levels were low at baseline, and many patients showed a weak response after nivolumab treatment (**Figure S2**). There were no obvious biomarkers in this set of data.

#### 3.4.5 Immuno-PGx analysis

Our data suggests that nonsynonymous SNVs in *IL7R* (rs6897932, C>T, OR = 8.98, 95% CI = 1.25–88.1 p = 0.029; rs1494555, G>A, OR = 6.00, 95% CI = 1.00–50.6, p = 0.049), and *PDCD1* (rs2227982, G>A, OR = 6.00, 95% CI = 1.00 – 50.6, p = 0.049) are closely associated with irAEs.

However, our evaluation of HLA typing revealed no significant predictive mutations in these genes for whether treatment efficacy or adverse events (data not shown).

## 4 Discussion

Our comprehensive evaluation of the biomarker data from the phase II nivolumab trial for thymic carcinoma revealed several biomarkers which may characterize long SD (+) samples, with this data revealing that these patients present with larger numbers of naïve/memory T cells prior to treatment and priming after nivolumab administration. In addition, our evaluation of the four irAE (+) cases, revealed that these patients presented with many effector T cells before the onset of irAE. Well-known biomarkers, including the expression of PD-L1 on tumor cells and tumor infiltrating lymphocytes (TILs, especially CD8^+^ tumor infiltrating T cells, and PD-1 positive Teff) (8–11) were not associated with therapeutic efficacy in this study. Interestingly, several nonsynonymous SNVs may explain both the treatment effects and adverse events catalogued in this study. In general, these data revealed that thymic carcinoma cases presented with low immunogenicity.

We revealed that large numbers of naïve/memory cells prior to treatment and priming after nivolumab administration were related to longer SD. Several studies have shown a correlation between the number of PBMCs and treatment effects in various cancers, including NSCLC (8), melanoma (9), gastric cancer (12), and malignant pleural mesothelioma (13). In addition, our results were also similar to those for other cancers with low immunogenicity, such as breast cancer (14) and malignant pleural mesothelioma (15). In breast cancer, atezolizumab, a PD-L1 antibody with ADCC activity against PD-L1 positive TNBC was shown to be effective (16), while malignant pleural mesothelioma achieved better treatment outcomes when treated using a combination of anti CTLA-4 and PD-1 antibodies (17). In addition, the ongoing NIVOTHYM study (nivolumab in patients with type B3 thymoma and thymic carcinoma, NCT03134118), revealed that the ORR was relatively low (12%) in the nivolumab monotherapy cohort, but the ORR for each histology has not yet been reported. Patients in the upcoming cohort will be treated with a combination of nivolumab and ipilimumab, which is expected to increase treatment efficacy.

Our evaluations also revealed that some of the patients in the irAE (+) group (#4, 10) presented with relatively high levels of several pro-inflammatory cytokines (IFNγ, IL-2, IL-7, TNFα, MIP1α, and MIP1β), whereas others (#13, 14) did not. Patients # 4 and # 10 experienced irAEs earlier than patients #13 and # 14, which might be explained by the fact that cytokine levels can be predictive for irAEs in malignant melanoma (10, 18, 19). However, irAEs in our study were not shown to be related to treatment effect.

FOXP3 expression in tumor area and stroma, CD204 expression in stroma, and MHC class I in tumor area were all low among long SD patients. Unexpectedly, lower PD-L1 expression was seen in the long SD (+) cases, and high CD4+ or CD8+ density appears not to be beneficial for longer SD. In PBMC analysis, the increased number of CD80+, Ki67+ naïve/memory T-cells, CD80+, PD-L1+ monocytes, and myeloid-derived suppressor cells (MDSCs) after nivolumab dose positively correlated with PFS. These data indicate the induction of T cell proliferation and activation and PD-L1 expression was induced in these cells. As a limited inference, the thymus is an organ responsible for cellular immunity, and its specificity might weaken anti-tumor response to existing immune cells.

This study is the first to explore the relationship between nonsynonymous germline SNVs, PFS, and irAEs in thymic carcinoma. Integrin alpha X (ITGAX) is also known as CD11c and is found on dendritic cells. SNVs in *ITGAX* (rs2230429) have been reported in some autoimmune diseases such as Bechet’s disease (20, 21) and systematic lupus erythematosus (22), but not in cancer. However, our data revealed that patients with homozygous mutations in *ITGAX* demonstrated a poorer prognosis than those without, which might be due to the associated decrease in the functioning of various antigen-presenting cells. *PDCD1* (rs2227982) has been reported to act as a predictive marker for PD-1 blockade (23), is associated with a decreased risk factor for breast cancer (24), and a possible risk factor of gastric cardia adenocarcinoma (25). However, our study is the first to find that *PDCD1* (rs2227982) is also a potential risk factor for both irAEs and PFS. Polymorphism rs6897932 in *IL7R* has been reported to be positively correlated with histopathological grade and lymph node metastasis in breast cancer (26), but no study has shown that genetic polymorphisms rs1494555, and rs6897932 are associated with an increased risk of irAEs, although these changes may suggest that changes in IL-7 may alter the balance between the membrane-bound (IL-7Rα) and soluble (sIL-7Rα) isoforms of this protein promoting excessive T cell growth and survival.

Despite the valuable insights offered by our evaluations our study did suffer from a few limitations. Owing to the interruption of the 2-stage design, only a limited number of patients were enrolled in this study. Some biomarkers with a clear trend should be interpreted with caution. PBMCs could not be processed at all centers and only one of the three patients with long SD underwent PBMC analysis, making it difficult to differentiate between long SD (+) and (-). In addition, although long SD is considered as a clinical benefit, the immune response may be different from that of partial response or complete response cases. To note, the outcomes (long SD, irAE) used to categorize the patients in this manuscript were not the objectives of the PRIMER study nor known markers of a drug’s activity. Genetic analysis of tumor cells, such as whole exome sequencing, RNA sequencing, methylation array, and copy number analyses, was not performed in this study because genomic testing for thymic carcinoma has been performed in previous reports (27, 28) and the known frequent genetic abnormalities were not previously shown to be associated with the therapeutic effect of nivolumab (27).

This study describes our comprehensive immune-related biomarker analysis of tissue and blood samples from patients participating in a phase II trial of nivolumab for thymic carcinoma. Our data are not generalizable to other ICIs, which have been successfully tested in the treatment of thymic carcinoma. We rather suggest that the therapeutic strategy for thymic carcinoma might require the activation of immune systems *via* a combination of immune checkpoint blockades.

## Data availability statement

'The original contributions presented in the study are publicly available. This data can be found here: https://humandbs.biosciencedbc.jp/en/hum0381-v1 [JGAS000588, JGAD000715].

## Ethics statement

This study was approved by the National Cancer Center Hospital (NCCH 2016-145), in accordance with the ethical guidelines set out in the Declaration of Helsinki.

## Author contributions

Concept and design, interpretation of data, and drafting of the manuscript, YK, SK, MY, and HH. Analysis, MO, SY, AH, HN, FH, and TSh. Pathology, NM and TN. Critical revision of the manuscript for important intellectual content, all co-authors. All authors contributed to the article and approved the submitted version.

## Funding

This work was supported by Japan Agency for Medical research and Development [grant number 17ck0106161h0003].

## Acknowledgments

The authors thank Ms. Sachiko Miura, Ms. Toshiko Chizu, and Ms. Sakaguchi Kina for their excellent technical assistance.

## Conflict of interest

SK reports grants and personal fees from Astra Zeneca, grants and personal fees from Pfizer, grants and personal fees from Boehringer Ingelheim, personal fees from Taiho, personal fees from Novartis, grants and personal fees from MSD, personal fees from Sumitomo Dainippon Pharma, grants and personal fees from Eisai, grants from Astellas, grants from Gilead Sciences, grants and personal fees from Ono Pharmaceutical Co., Ltd., personal fees from Bristol-Myers Squibb, grants and personal fees from REGENERON, personal fees from Rakuten Medical, grants from PACT Pharma, grants from Takara Bio Inc., personal fees from GSK, grants and personal fees from Daiichi-Sankyo, grants and personal fees from Chugai, personal fees from ImmuniT Research Inc., grants from AMED(Japan Agency for Medical Research and Development), grants from JSPS(Japan Society for the Promotion of Science), personal fees from PMDA(Pharmaceuticals and Medical Devices Agency), outside the submitted work. SY reports grants from Nippon Boheringer Ingelheim, outside the submitted work. AH reports Grants or contracts from Eisai, Healios, Eli Lilly, Daiichi Sankyo, Chordia Therapeutics, Konica Minolta, outside the submitted work. TSe reports Grants or contracts from Abbvie, Chugai Pharmaceutical, Daiichi Sankyo, Eli Lilly Japan, Kissei Pharmaceutical, MSD, Novartis Pharma. Pfizer Japan, Takeda Pharmaceutical, and Payment of honoraria from AstraZeneca, Bristol-Myers Squibb, Chugai Pharmaceutical, Covidien Japan, Daiichi Sankyo, Eli Lilly Japan, Kyowa Hakko Kirin, MSD, Mochida Pharmaceutical, Nippon Boehringer Ingelheim, Novartis Pharma, Ono Pharmaceutical, Pfizer Japan, Taiho Pharmaceutical, Takeda Pharmaceutical, Towa Pharmaceutical, and other interests from Precision Medicine Asia, outside the submitted work. SU reports Payment of honoraria from Chugai Pharmaceutical, outside the submitted work. YH reports Payment of honoraria from AstraZeneca, Chugai Pharmaceutical, Ono Pharmaceutical, Bristol-Myers Squibb, Eisai, outside the submitted work. MS reports Grants or contracts from Ono, Bristol Myers Squibb, Chugai, MSD, IQVIA, EPS, Janssen, Amgen, Taiho, Pfizer, Abbvie, Daiichi-Sankyo, Takeda, Eisai, and Payment of honoraria from Ono, Bristol Myers Squibb, Chugai, MSD, AstraZeneca, Eli Lilly, Taiho, Pfizer, Novartis, Takeda, Merck, outside the submitted work. MN reports grants and personal fees from Ono Pharmaceutical, grants and personal fees from Bristol Myers Squibb, grants and personal fees from Pfizer, grants and personal fees from Chugai Pharmaceutical, grants and personal fees from Eli Lilly, grants and personal fees from Taiho Pharmaceutical, grants and personal fees from AstraZeneca, personal fees from Boehringer-Ingelheim, grants and personal fees from MSD, grants and personal fees from Novartis, personal fees from Merck Biopharma, grants and personal fees from Daiichi Sankyo, grants and personal fees from Takeda Pharmaceutical Company Limited, personal fees from Teijin Pharma Limited., personal fees from AbbVie, outside the submitted work. TK reports Consulting fees from Chugai Pharmaceutical Co., AstraZeneca, Ono Pharmaceutical Co., Pfizer Japan, Daiichi-Sankyo, Bayer, Abbvie, and Payment of honoraria from Chugai Pharmaceutical Co., AstraZeneca, Eli Lilly Japan, Taiho Pharmaceutical Co., Bristol-Myers Squibb, Ono Pharmaceutical Co., MSD, Pfizer Japan, Kyowa Hakko Kirin, Nippon Beohringer Ingelheim, Merck Biophama, Nippon Kayaku, Novartis, Daiichi-Sankyo, Takeda Pharmaceutical Co., Bayer, Sawai, outside the submitted work. TH reports Payment or honoraria from ONO Pharmaceutical, Bristol-Meyers Squibb, MSD, outside the submitted work. YO is an editorial board member, and reports Grants or contracts from AstraZeneca, Chugai, Lilly, ONO, BMS, Kyorin, Dainippon- Sumitomo, Pfizer, Taiho, Novartis, Takeda, Kissei, Daiichi-Sankyo, Janssen, LOXO, and Payment or honoraria from AstraZeneca, Chugai, Eli Lilly, ONO, BMS,Boehringer Ingelheim, Bayer, Pfizer, MSD, Taiho, Nippon Kayaku, Kyowa Hakko Kirin, and Payment for expert testimony from AstraZeneca, Chugai, ONO, BMS, Kyorin, Celltrion, Amgen, Nippon Kayaku, Boehringer Ingelheim, AnHeart Therapeutics Inc., and Leadership or fiduciary role in JSMO, JLCS, JCOG, outside the submitted work. HH reports Grants or contracts from MSD, Abbvie, AstraZeneca, BMS, Ono, Merck Biophama, Daiichi-Sankyo, Janssen, Genomic Helath, Chugai, Roche, and Novartis, and Payments or honoraria from AstraZeneca, MSD, Eli Lilly, Ono, BMS, Chugai, Roche, Kyowa-Kirin, and Novartis, and Participation on an Advisory board for AstraZeneca, Eli Lilly, Chugai, Roche, ONO, BMS, and MSD, outside the submitted work.

The remaining authors declare that the research was conducted in the absence of any commercial or financial relationships that could be construed as a potential conflict of interest.

## Publisher’s note

All claims expressed in this article are solely those of the authors and do not necessarily represent those of their affiliated organizations, or those of the publisher, the editors and the reviewers. Any product that may be evaluated in this article, or claim that may be made by its manufacturer, is not guaranteed or endorsed by the publisher.

## Supplementary material

Supplementary Figure 1 The ratio of C3D1 values to pre-values of PBMC count in each patient and correlation with PFS.

Supplementary Figure 2 Serum concentrations trend of each cytokine.

Supplementary Figure 3 KM curve for PFS in each SNV group: ITGAX (hetero vs homo, HR 0.27, 95% CI 0.069–1.12, p=0.067), PDCD1 (wild+hetero vs homo, HR 0.20, 95% CI 0.039–1.02, p=0.053).

Supplementary Table 1 Antibody list for Multiplex fluorescence immunohistochemistry (mFIHC).

Supplementary Table 2 Antibody list for Flow cytometer.

Supplementary Table 3 List for NCC immuno pharmacogenomic (PGx) analysis panel.
