# Supplementary figures and images for: Comprehensive biomarker analysis from phase II study of nivolumab in patients with thymic carcinoma
Source: Front Oncol. 2023 Jan 9;12:966527. doi: 10.3389/fonc.2022.966527 (PMC9869613; doi:10.3389/fonc.2022.966527)

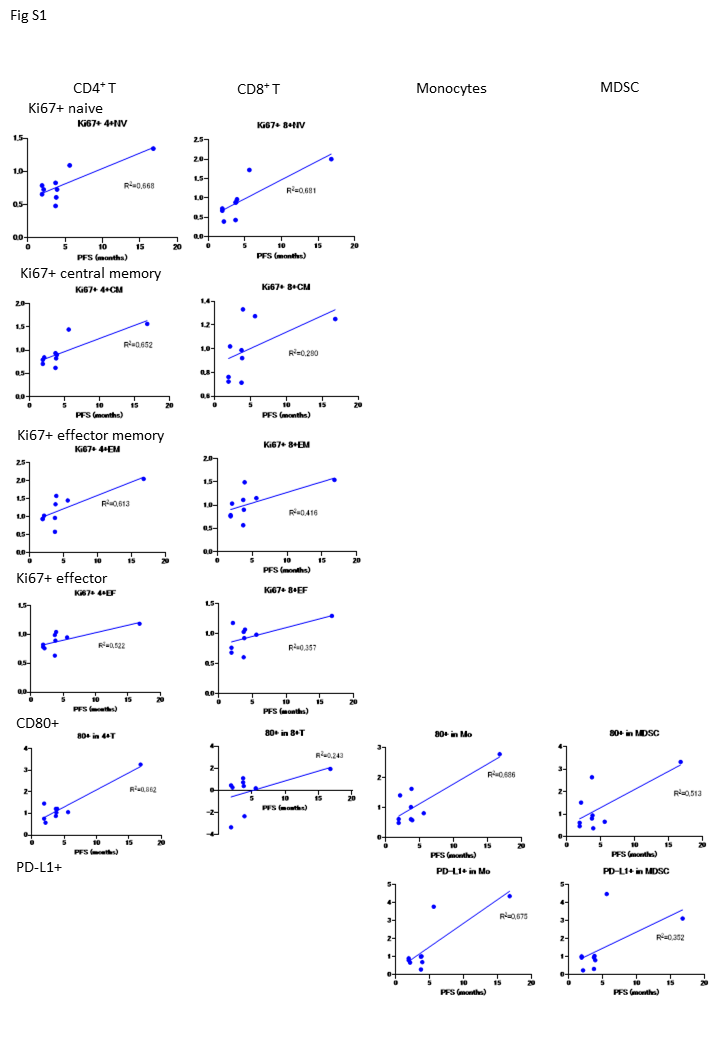

Supplement: Supplementary Figure 1 — The ratio of C3D1 values to pre-values of PBMC count in each patient and correlation with PFS. [file Image_1.tif]

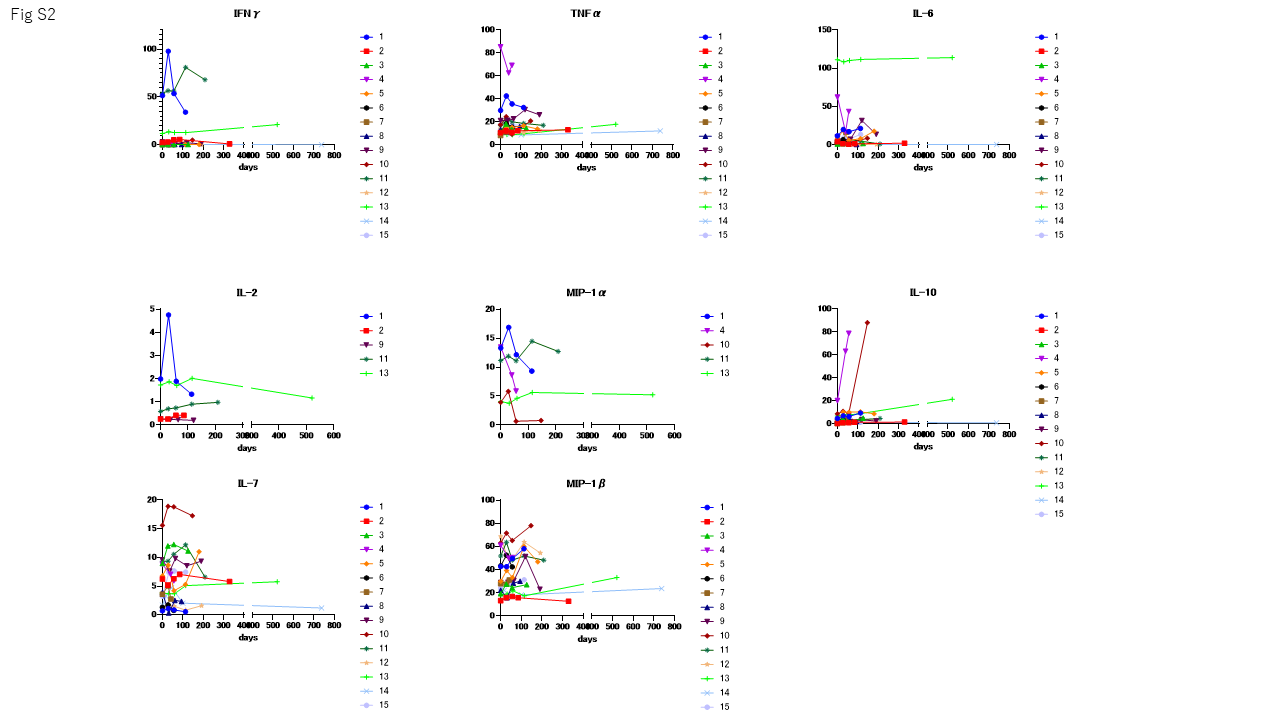

Supplement: Supplementary Figure 2 — Serum concentrations trend of each cytokine. [file Image_2.tif]

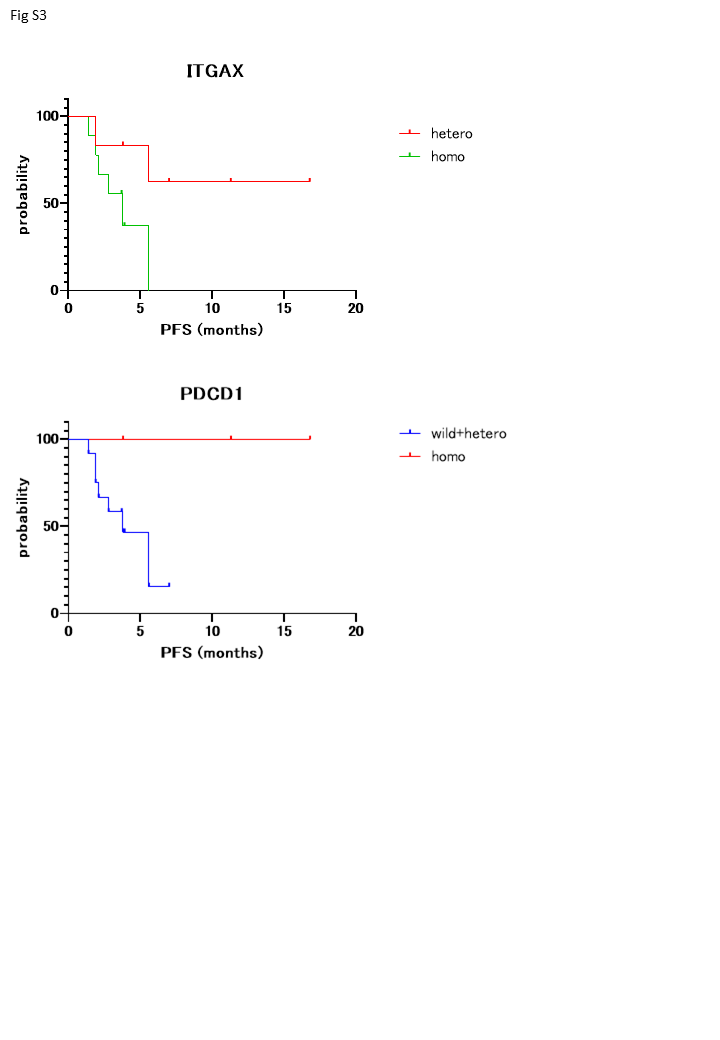

Supplement: Supplementary Figure 3 — KM curve for PFS in each SNV group: ITGAX (hetero vs homo, HR 0.27, 95% CI 0.069–1.12, p=0.067), PDCD1 (wild+hetero vs homo, HR 0.20, 95% CI 0.039–1.02, p=0.053). [file Image_3.tif]
